# Mapping the structure of neural states associated with conscious experience

**DOI:** 10.3389/fnhum.2026.1784365

**Published:** 2026-04-09

**Authors:** Francis G. Smith

**Affiliations:** Independent Researcher, Baltimore, MD, United States

**Keywords:** neural correlates of consciousness, neural manifolds, neural state space, perturbational complexity, representational geometry

## Abstract

A central challenge in consciousness research concerns the relationship between neural activity and conscious experience. While decades of work have identified numerous neural correlates of consciousness, these findings increasingly indicate that activation magnitude, localization, or stimulus processing alone are insufficient to account for awareness. What remains less clearly articulated is how neural activity is organized in association with conscious experience. In this review, we synthesize empirical findings that bear on the structure of neural states correlated with conscious experience. Drawing on research in neural correlates of consciousness, representational similarity analysis, neural manifold studies, perturbational approaches, and canonical sensory systems, we examine how neural activity is organized in state space across conscious and unconscious conditions. Across paradigms and measurement modalities, conscious experience is consistently associated with neural states that are restricted to admissible configurations, organized within low-dimensional subspaces or manifolds, structured by meaningful geometric and topological relationships, and dynamically accessible under perturbation. We show that distances in neural representational space track experiential similarity, that categorical perceptual distinctions correspond to clustering and boundaries in neural state space, and that perturbational measures distinguish accessible experiential states from inactive or fragmented configurations. Rather than advancing a specific theory of consciousness, this review provides a unifying structural synthesis that clarifies empirically grounded constraints on the neural organization associated with conscious experience.

## 1 Introduction

### 1.1 Motivation: beyond neural activity toward structural descriptions

A central aim of contemporary neuroscience is to characterize the neural states associated with conscious experience. Decades of experimental work have established that conscious perception, imagery, and thought reliably correlate with specific patterns of neural activity across distributed brain networks (Crick and Koch, 2003; Koch et al., 2016). However, it has become increasingly clear that the mere presence of neural activation is insufficient to account for conscious experience. Neural activity can occur in the absence of reported experience, and conversely, similar levels of activity can correspond to qualitatively distinct experiences (Aru et al., 2012).

This dissociation has been demonstrated across a wide range of paradigms, including binocular rivalry, visual masking, blindsight, anesthesia, sleep, and disorders of consciousness (Logothetis, 1998; Tong et al., 1998; Tamietto and Koch, 2008). In these cases, neural responses may persist despite the absence of conscious awareness, or conscious experience may fluctuate without corresponding changes in sensory input. Such findings indicate that the relationship between neural activity and conscious experience cannot be captured solely in terms of activation magnitude, localization, or task engagement.

**Figure 1:**
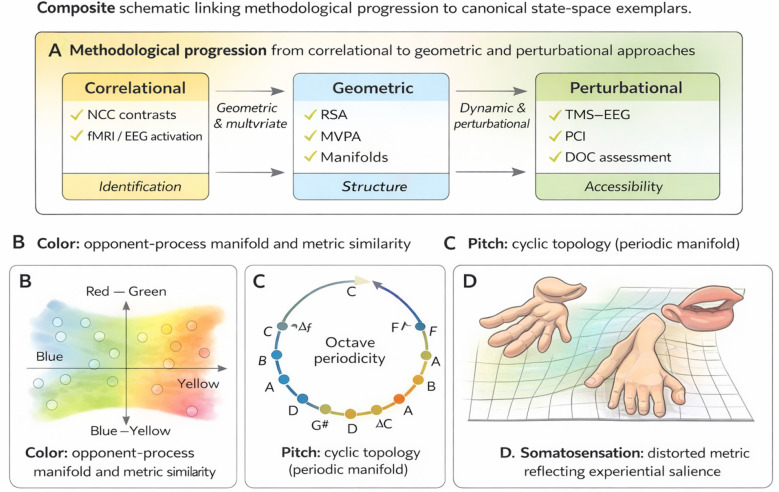
Composite schematic linking methodological progression to canonical state-space exemplars. **(A)** Consciousness research has progressed from correlational paradigms that identify neural states associated with reported experience to geometric and multivariate approaches that reveal organized, low-dimensional state-space structure, and to perturbational methods that directly probe accessibility, integration, and stability. **(B–D)** Canonical sensory domains illustrate how qualitative experience corresponds to structured neural state spaces: **(B)** color perception organized along opponent-process dimensions, **(C)** auditory pitch exhibiting cyclic topology, and **(D)** somatosensory experience characterized by distorted metric structure reflecting experiential salience. The figure is schematic and intended to summarize convergent empirical findings rather than depict a specific mechanistic or theoretical model.

In response, recent research has increasingly emphasized the organization of neural states rather than their presence or absence. Multivariate decoding, representational similarity analysis, and neural manifold approaches reveal that neural activity occupies structured regions of high-dimensional state space, with systematic geometric relationships between patterns (Kriegeskorte et al., 2008; Cunningham and Yu, 2014; Vyas et al., 2020). Separately, perturbational approaches demonstrate that conscious states differ from unconscious ones in their accessibility, integration, and response to external perturbation, even when baseline activity appears comparable (Casali et al., 2013; Rosanova et al., 2018) (Figure 1).

Taken together, these results suggest that conscious experience is associated not simply with neural activity, but with restricted, structured, and accessible configurations of neural state space. Despite this convergence, the relevant findings remain fragmented across experimental traditions and theoretical frameworks. What is currently lacking is a unified synthesis focused specifically on the structural properties of neural states correlated with conscious experience—properties such as geometry, topology, dimensionality, stability, and accessibility—that recur across paradigms and measurement modalities.

This review aims to address that gap by surveying and integrating empirical results that bear on the structural organization of neural states associated with conscious experience, without presupposing a particular explanatory theory.

### 1.2 Scope, definitions, and exclusions

The focus of this review is descriptive and integrative rather than mechanistic or ontological. We examine experimental and computational studies that investigate the structure of neural states correlated with reported conscious experience, including work on neural correlates of consciousness, representational geometry, perturbational complexity, and canonical sensory spaces. Throughout, the term *neural state* refers to a pattern of activity across neural populations at a given time or over a defined temporal window, as operationalized by the measurement modality in question (Cunningham and Yu, 2014).

Importantly, this review does not propose a new theory of consciousness, nor does it seek to adjudicate among existing theoretical frameworks such as global workspace, integrated information, higher-order, or predictive processing accounts (Baars, 1988; Dehaene and Changeux, 2011; Tononi, 2004; Friston, 2010). Where such theories are referenced, they are treated as sources of experimental paradigms or analytic tools rather than as explanatory commitments. The review also does not attempt to resolve the philosophical “hard problem” of consciousness or to offer an account of why particular neural states are associated with subjective experience (Overgaard, 2018).

Additionally, we restrict attention to empirical results that directly bear on the organization of neural states associated with experience, rather than on purely behavioral measures, introspective reports in isolation, or speculative mappings from artificial systems to consciousness. While animal studies are included where they illuminate structural principles of neural organization, the primary emphasis is on human data, particularly where subjective report or clinical assessment is available (Koch et al., 2016; Boly et al., 2017).

By clearly delimiting its scope, this review aims to provide a neutral synthesis of what is currently known about the structural properties of neural states correlated with conscious experience, and to clarify which aspects of this structure are empirically established, which remain uncertain, and which represent promising directions for future investigation (Table 3).

### 1.3 Scope of the explanandum

Throughout this review, the term “consciousness” is used in a deliberately restricted empirical sense. The focus is on reportable, wakeful conscious states associated with perceptual awareness and experiential content in adult human subjects under typical conditions of arousal. The aim is not to provide a comprehensive account of phenomenal ontology, nor to equate consciousness with intelligence, cognitive control, or general information processing.

Many neural processes involved in perception, learning, and decision-making occur without conscious access and therefore fall outside the explanandum considered here. Instead, the present analysis concerns neural states that reliably co-occur with subjectively reportable experience, as indexed by contrastive paradigms, perturbational approaches, and level-of-consciousness manipulations (e.g., wakefulness vs. anesthesia).

By sharpening the explanandum in this way, the structural constraints discussed below should be understood as characterizations of empirically accessible conscious states rather than as exhaustive accounts of subjectivity.

Table 1 summarizes the principal paradigms and datasets that anchor the structural synthesis developed in Sections 2–8.

**Table 1:** Representative data sources and experimental paradigms for studying structural properties of neural states associated with conscious experience.

Data source paradigm	Modality	Key empirical finding and structural insight	Relevance to qualia structure
Neural correlates of consciousness (contrastive paradigms)	fMRI, EEG, MEG, iEEG	Conscious perception corresponds to a subset of task-relevant activity; many active states are not consciously experienced. *Structural property:* State restriction/selection	Indicates admissible vs. non-admissible neural configurations
Binocular rivalry and masking	fMRI, single-unit, EEG	Neural activity tracks perceptual alternations despite constant sensory input. *Structural property:* State switching and boundaries	Structured transitions between experiential states
No-report paradigms	fMRI, EEG	Posterior cortical activity predicts perceptual content independent of motor report. *Structural property:* Decoupling report from experience	Structural correlates rather than behavioral artifacts
Representational similarity analysis	fMRI, MEG	Distances in neural state space correlate with phenomenological similarity. *Structural property:* Metric geometry	Geometric organization of experiential similarity
Multivariate pattern analysis	fMRI	Experiential categories occupy separable regions of neural space. *Structural property:* Clustering and separability	Categorical topology of qualia
Neural manifold studies	Single-unit, Calcium imaging, fMRI	High-dimensional neural activity collapses onto low-dimensional manifolds. *Structural property:* Dimensional reduction	Low-dimensional organization of experience
Human Connectome Project	fMRI, MEG	Brain activity occupies constrained regions across tasks and individuals. *Structural property:* Global state organization	Large-scale structured state space
Perturbational Complexity Index (PCI)	TMS-EEG	Conscious states show complex, integrated responses to perturbation. *Structural property:* Accessibility and integration	Accessible vs. inaccessible regions of state space
Anesthesia and sleep datasets	EEG, fMRI	Loss of consciousness correlates with reduced integration and reachability. *Structural property:* State collapse and fragmentation	Breakdown of experiential structure
Disorders of consciousness	EEG, TMS-EEG, fMRI	Neural activity may persist without experiential accessibility. *Structural property:* Boundary conditions	Separates activation from admissibility
Color perception datasets	fMRI, psychophysics	Color experience maps onto opponent-process geometry. *Structural property:* Continuous manifolds	Canonical structured qualia space
Auditory pitch perception	Single-unit, fMRI	Pitch encoded along continuous, cyclic manifolds. *Structural property:* Periodic topology	Non-trivial experiential topology
Somatosensory mapping	Electrical stimulation, fMRI	Cortical magnification reflects experiential salience. *Structural property:* Distorted metric structure	Non-uniform experiential geometry

Focus: geometry, topology, dimensionality, and accessibility.

## 2 Neural correlates of consciousness as state restriction

### 2.1 Contrastive paradigms and the identification of conscious states

Research on the neural correlates of consciousness (NCC) has traditionally relied on contrastive paradigms, in which neural activity associated with conscious perception is compared to activity measured under closely matched conditions lacking reported experience (Crick and Koch, 2003; Dehaene and Naccache, 2001). Common examples include binocular rivalry, visual masking, attentional blink, and perceptual threshold paradigms. In these settings, sensory input and task demands can be held constant while subjective experience varies, allowing neural differences to be attributed to conscious perception rather than stimulus properties alone.

Across such paradigms, a consistent finding is that conscious perception does not correspond to a simple increase in overall neural activity. Instead, neural responses associated with conscious experience involve specific patterns of distributed activation, often within posterior sensory and associative cortices (Logothetis, 1998; Tong et al., 1998). Comparable levels of activity may be observed under conditions where experience is absent or degraded, indicating that activity magnitude alone is insufficient to account for conscious access (Aru et al., 2012).

These results already imply a form of state restriction: among the many neural states compatible with sensory processing and task performance, only a subset reliably corresponds to conscious experience.

### 2.2 Dissociations between neural activity and experience

Strong evidence for state restriction arises from dissociations in which neural activity persists despite the absence of conscious experience, or in which experience changes without commensurate changes in sensory input. Blindsight provides a canonical example, in which patients with lesions to primary visual cortex retain the ability to discriminate visual stimuli without visual awareness (Tamietto and Koch, 2008). Neuroimaging and electrophysiological studies demonstrate that substantial visual processing remains intact in such cases, yet the resulting neural states do not support conscious vision.

Similar dissociations are observed during general anesthesia, deep non-rapid eye movement sleep, and certain disorders of consciousness. In these conditions, large-scale neural activity and sensory responses may persist, but conscious experience is diminished or absent (Koch et al., 2016). Conversely, during phenomena such as binocular rivalry or perceptual switching, subjective experience alternates despite constant external stimulation and relatively modest changes in overall neural activation (Logothetis, 1998).

Together, these findings demonstrate that conscious experience is not determined solely by whether neural activity is present, but by whether neural activity occupies particular configurations that support experiential access.

### 2.3 No-report paradigms and the role of behavioral confounds

A persistent concern in NCC research is the potential confounding influence of behavioral report. Tasks requiring explicit responses may engage additional cognitive processes, including attention, decision-making, and motor preparation, which can obscure the neural correlates of experience itself (Aru et al., 2012). To address this issue, no-report paradigms have been developed in which perceptual content is inferred from physiological markers, eye movements, or stimulus-driven signatures rather than explicit reports.

Results from no-report studies largely converge with those obtained from traditional paradigms, reinforcing the conclusion that conscious experience corresponds to specific patterns of neural organization rather than to reporting behavior (Boly et al., 2017). In particular, posterior cortical activity often tracks perceptual content even when reports are absent, whereas frontal activity appears more closely related to task demands and introspection (Koch et al., 2016).

From a structural perspective, these findings strengthen the interpretation that conscious experience is associated with admissible neural states defined by their internal organization, not by downstream behavioral outputs.

### 2.4 Minimal neural substrates and admissible state space

An important goal of NCC research has been the identification of minimal neural substrates sufficient for conscious experience. While consensus has not been reached on precise anatomical boundaries, converging evidence suggests that conscious perception depends on coordinated activity within restricted cortical networks, particularly within posterior sensory and associative regions (Boly et al., 2017).

Crucially, this coordination appears to be selective. Neural activity outside these networks, or activity lacking appropriate integration and organization, does not reliably support experience, even when it is functionally relevant to behavior (Dehaene and Changeux, 2011). This observation reinforces the view that conscious experience corresponds to a constrained subset of possible neural configurations.

Rather than defining consciousness in terms of specific regions or signals, the accumulated evidence from NCC studies is naturally interpreted in terms of admissibility: among the many neural states generated by ongoing brain dynamics, only certain configurations are compatible with conscious experience.

### 2.5 Reinterpreting neural correlates of consciousness in structural terms

The classical formulation of the neural correlates of consciousness (NCC), following Crick and Koch (1990), seeks the minimal neural mechanisms jointly sufficient for a specific conscious percept or experience. This formulation has guided contrastive paradigms and localization efforts for several decades.

The structural perspective advanced in this review does not discard the NCC framework, but reframes its interpretation. Rather than identifying NCC with a localized minimal sufficient mechanism, the present synthesis suggests that NCC may be more fruitfully understood as the restriction of neural activity to a particular region of state space characterized by geometric organization, low-dimensional embedding, accessibility, and constrained transitions.

In this view, what is traditionally described as an NCC may correspond not to a discrete neural module or activation locus, but to the occupation and traversal of a structurally constrained manifold. Localized activity patterns remain important, but their relevance derives from their participation in a restricted and dynamically accessible state-space geometry.

This reinterpretation remains fully compatible with empirical NCC methodology. Contrastive paradigms, perturbational approaches, and decoding analyses can be understood as probing the boundaries and properties of such restricted regions. The proposal therefore does not replace the NCC concept, but situates it within a broader structural account of neural organization.

### 2.6 Summary: NCC as evidence for structural constraints

Viewed collectively, NCC research supports a structural interpretation of the neural basis of conscious experience. Consciousness does not track neural activity in a generic or global manner, but instead corresponds to restricted configurations characterized by specific patterns of coordination, integration, and accessibility (Koch et al., 2016).

In this sense, NCC findings motivate a shift in emphasis from identifying isolated neural correlates to characterizing the structure of neural state space itself. The key empirical question becomes not which neural events occur during conscious experience, but which neural states are admissible as experiential states and how these states are organized relative to the broader landscape of neural dynamics.

This geometric framing provides a natural bridge to approaches that explicitly analyze the geometry and dimensionality of neural representations, which are the focus of the next section.

## 3 Neural state-space geometry and representational structure

### 3.1 High-dimensional neural activity and state-space formulation

Neural activity arises from the coordinated dynamics of large populations of neurons, giving rise to activity patterns that are intrinsically high-dimensional. At any given moment, the state of a neural system can be represented as a point in a multidimensional space whose axes correspond to neural variables such as firing rates, local field potentials, or population-level signals measured by neuroimaging techniques (Cunningham and Yu, 2014). Over time, neural dynamics trace trajectories through this state space.

This formulation has proven useful across multiple domains of neuroscience, including perception, motor control, and memory, as it provides a unified framework for comparing neural states across conditions and time (Vyas et al., 2020). For the study of conscious experience, the state-space perspective allows neural states associated with different experiential contents or levels of awareness to be analyzed in terms of their relative positions, distances, and transitions within a common representational landscape. Consistent with the scope defined above, this refers to reportable perceptual and wakeful states in human subjects.

From this perspective, the central question is not whether a particular neural signal is present, but how neural states associated with conscious experience are organized relative to other states generated by ongoing brain dynamics.

### 3.2 Dimensionality reduction and neural manifolds

Although neural state spaces are high-dimensional in principle, empirical studies consistently find that neural activity occupies a much lower-dimensional subspace. Dimensionality reduction techniques such as principal component analysis, factor analysis, and nonlinear embedding methods reveal that neural dynamics often lie on structured manifolds embedded within the larger state space (Cunningham and Yu, 2014; Gallego et al., 2017).

These manifolds capture dominant modes of population activity and provide compact descriptions of neural dynamics across tasks and conditions. In sensory systems, for example, neural responses to a wide range of stimuli can often be described by a small number of latent dimensions corresponding to perceptually salient features (Cichy et al., 2014). Similar low-dimensional structure has been observed in higher-order cortical regions and at the level of large-scale brain networks (Vyas et al., 2020).

For conscious experience, the relevance of neural manifolds lies in their capacity to constrain and organize possible neural states. If neural activity relevant to experience is confined to specific manifolds, then conscious states correspond not to arbitrary points in state space, but to locations within these structured subspaces.

It should be noted that foundational insights into population-level geometry originate in non-human animal research, including primate studies of orientation and color manifolds in visual cortex and whole-brain calcium imaging in rodents. While such studies lack direct subjective report, they have been instrumental in establishing the methodological tools used to characterize neural manifolds in human consciousness research. The present review focuses on paradigms involving reportable experience but builds upon this broader empirical foundation.

### 3.3 Representational similarity and metric structure

Beyond dimensionality, the geometry of neural representations provides a powerful link between neural activity and experiential content. Representational similarity analysis (RSA) and related multivariate approaches quantify the distances between neural activity patterns evoked by different stimuli or conditions, allowing neural representational geometry to be compared with behavioral or phenomenological measures (Kriegeskorte et al., 2008).

Across sensory modalities, RSA studies demonstrate that distances in neural representational space often track perceptual similarity judgments: stimuli that are experienced as similar evoke nearby neural states, whereas perceptually distinct stimuli occupy more distant regions of state space (Haxby et al., 2001; Kamitani and Tong, 2005). This correspondence suggests that experiential similarity is reflected in the metric structure of neural representations.

Crucially, this relationship emerges at the level of population geometry rather than from single units or localized activations. Neural representations supporting conscious experience are distributed and relational, reinforcing the importance of state-space structure over localized signals (Norman et al., 2006; Haynes and Rees, 2006).

### 3.4 Clustering, boundaries, and categorical organization

Neural state-space analyses further reveal that representations often form clusters corresponding to perceptual or cognitive categories. These clusters are not always sharply separated, but may be connected by transitional regions reflecting gradual changes in experience. Such organization has been observed in visual object categories, color perception, and auditory representations (Cichy et al., 2014; Haxby et al., 2001).

The presence of clustering and boundaries suggests that experiential categories are grounded in the structure of neural representations rather than imposed arbitrarily. From a state-space perspective, categorical perception corresponds to regions of relative stability within neural state space, separated by boundaries where small changes in neural activity can lead to large changes in experience.

This organization highlights the coexistence of continuity and discreteness in experiential structure, and cautions against overly simplistic mappings between neural signals and phenomenological categories.

### 3.5 State trajectories and experiential transitions

State-space approaches also enable the analysis of how neural activity evolves over time, tracing trajectories through representational space as perception, attention, or conscious state changes. Rather than focusing solely on static representations, recent work emphasizes the dynamics of population activity and the constraints governing transitions between states (Vyas et al., 2020).

Phenomena such as perceptual switching during binocular rivalry, changes in attentional focus, or transitions between wakefulness and sleep can be analyzed as movements between regions of state space. These trajectories often follow characteristic paths, suggesting that not all state transitions are equally accessible (Cunningham and Yu, 2014).

From a structural standpoint, these findings reinforce the view that conscious experience is associated with regions of state space that are not only geometrically organized but also dynamically traversable in constrained ways.

### 3.6 Formal illustration of state-space restriction

To clarify what is meant by “geometry” and “restriction” in neural state space, consider a neural population activity vector


$$\text{x}\left(t\right)\in {\mathbb{R}}^{N},$$

where *N* indexes recorded neurons or features at time *t*. In many empirical studies, high-dimensional activity trajectories are found to lie within a lower-dimensional subspace or manifold


$$M\subset {\mathbb{R}}^{N},$$

estimated using dimensionality-reduction or manifold-learning techniques such as principal component analysis, factor analysis, or nonlinear embedding methods (Cunningham and Yu, 2014; Gao et al., 2017).

Under many conscious perceptual conditions, neural trajectories tend to remain confined to structured, low-dimensional manifolds characterized by smooth transitions and stable relational geometry among activity patterns. By contrast, unconscious or anesthetized states may exhibit either excessive restriction (collapse toward fixed points or highly stereotyped trajectories) or fragmentation (loss of coherent manifold structure) (Mashour et al., 2020; Alkire et al., 2008).

Within this review, “state-space restriction” refers to the empirical observation that conscious processing occupies a constrained region


R⊂M,

defined not solely by activation magnitude but by the geometry of allowable transitions among states. Accessibility and constrained transitions may then be operationalized in terms of reachability and stability within this restricted manifold, measurable through perturbational paradigms or transition probability analyses (Casali et al., 2013; Schartner et al., 2017).

This schematic formalization is intended to clarify terminology rather than to privilege any specific analytic method. The structural claims advanced here concern recurring empirical features of neural state geometry, not a particular dimensionality-reduction technique.

### 3.7 Sampling within restricted state spaces

An additional implication of state-space restriction is that conscious mental processing need not correspond to a single fixed neural configuration. Instead, empirical manifold analyses suggest that activity trajectories may traverse a constrained region of a low-dimensional subspace while preserving geometric relations among states (Cunningham and Yu, 2014; Gao et al., 2017). In this sense, conscious processing can be understood as sampling within a restricted region of neural state space rather than occupying a static attractor.

Such sampling provides a natural explanation for robustness under biological variability. Distinct microstates may differ at the level of individual neurons or fine-scale activation patterns while remaining geometrically equivalent at the population level. Representational similarity structure and transition topology may therefore remain stable even as the precise neural instantiation fluctuates (Kriegeskorte et al., 2008; Mante et al., 2013).

Importantly, this interpretation remains entirely compatible with empirical NCC research. The structural features identified in this review—restriction, low-dimensional embedding, accessibility, and constrained transitions—are not tied to singular neural states but to regions of state space that preserve relational organization across time. Conscious experience, on this view, corresponds to trajectories that remain within such constrained manifolds, rather than to isolated activation patterns.

### 3.8 Geometry as a structural signature of experience

Collectively, findings from representational geometry, dimensionality reduction, and neural manifold analyses suggest that neural states associated with conscious experience are organized within structured, low-dimensional regions of state space. These regions exhibit metric relationships that track experiential similarity, clustering that reflects categorical organization, and constrained trajectories that shape experiential transitions.

Rather than identifying specific neural signals as discrete markers of consciousness, this body of work supports a structural characterization: conscious processing reliably occurs within particular regions of an organized representational geometry. This perspective complements classical NCC findings and motivates approaches that probe the accessibility and transition structure of these states through perturbation, which we consider in the next section.

## 4 Perturbational approaches and accessibility of neural states

### 4.1 Motivation: probing beyond spontaneous activity

Correlational approaches and representational geometry characterize where neural states associated with conscious experience reside within neural state space, but they do not by themselves establish whether such states are dynamically accessible or capable of sustaining experience. Neural activity with similar representational structure may occur under both conscious and unconscious conditions, particularly when assessed using baseline activation or local encoding measures (Koch et al., 2016).

Perturbational approaches address this limitation by actively displacing the neural system from its current state and measuring the ensuing spatiotemporal response. By examining how activity propagates, differentiates, and reintegrates following perturbation, these methods probe properties of accessibility, integration, and stability that are not apparent from spontaneous activity alone (Massimini et al., 2012).

### 4.2 TMS-EEG and the perturbational complexity index

One of the most influential perturbational frameworks combines transcranial magnetic stimulation (TMS) with high-density electroencephalography (EEG). In this paradigm, brief magnetic pulses perturb localized cortical regions, and the resulting EEG responses are analyzed to quantify the complexity of the brain's reaction.

The perturbational complexity index (PCI) was developed to capture the degree to which the evoked response is both spatially differentiated and temporally integrated (Casali et al., 2013). Empirically, PCI reliably distinguishes conscious states, such as wakefulness and dreaming, from unconscious states including deep non-rapid eye movement sleep, general anesthesia, and certain disorders of consciousness. Importantly, this distinction holds even in the absence of sensory input or behavioral responsiveness, indicating that PCI does not simply reflect perception or motor output.

From a structural standpoint, high PCI values indicate that perturbations engage widespread, differentiated neural dynamics, whereas low PCI values correspond to responses that are either locally confined or globally stereotyped. These differences suggest that conscious states occupy regions of neural state space that are dynamically accessible and capable of sustaining complex internal interactions.

### 4.3 Accessibility and reachability of neural state space

Perturbational studies provide direct evidence that not all neural states are equally reachable or revisitable. In unconscious states, perturbations tend to elicit responses that rapidly decay, fragment, or collapse into highly stereotyped patterns (Rosanova et al., 2018). In contrast, during conscious states, identical perturbations generate extended, differentiated trajectories that traverse multiple regions of neural state space before returning to baseline.

This contrast can be interpreted in terms of accessibility: conscious states reside in regions of state space that permit widespread propagation and re-entrant dynamics, whereas unconscious states are confined to regions with reduced connectivity or limited dynamical repertoire. Crucially, accessibility is not reducible to overall activity level, as comparable levels of neural activation may be observed across conscious and unconscious conditions (Schartner et al., 2015).

### 4.4 Perturbation, integration, and loss of consciousness

Changes in global brain state induced by sleep, anesthesia, or brain injury provide natural contexts for examining perturbational responses. Across these conditions, the loss of consciousness is accompanied by a marked reduction in the complexity and integration of perturbation-evoked activity (Koch et al., 2016; Massimini et al., 2012). Neural responses may remain robust at local scales, but their capacity to engage distributed networks is diminished.

These findings suggest that the loss of consciousness involves a collapse of accessible regions of neural state space rather than a simple suppression of neural activity. Recovery of consciousness, by contrast, is associated with the re-emergence of integrated and differentiated perturbational responses, consistent with a restoration of accessible state-space structure (Rosanova et al., 2018).

### 4.5 Clinical and noninvasive extensions

Perturbational measures have been applied in clinical contexts to assess residual consciousness in patients with disorders of consciousness. In some cases, preserved perturbational complexity has been observed despite minimal behavioral responsiveness, highlighting dissociations between overt behavior and experiential capacity (Boly et al., 2017).

Converging evidence also comes from intracranial electrical stimulation studies conducted during neurosurgical procedures, where stimulation can elicit specific perceptual experiences or disrupt ongoing experience depending on the site and timing of perturbation. These results further support the interpretation that experiential states correspond to selectively accessible regions of neural state space.

### 4.6 Summary: accessibility as a structural criterion

Perturbational approaches demonstrate that conscious experience is associated with neural states that are not only geometrically organized, but also dynamically accessible, integrated, and stable under perturbation. These properties distinguish conscious states from unconscious ones even when baseline activity or representational structure appears similar.

From a structural perspective, measures such as PCI identify which regions of neural state space are capable of sustaining the rich dynamics associated with conscious experience. Accessibility thus emerges as a key empirical criterion, complementing evidence from NCC studies and representational geometry. Together, these findings reinforce the view that conscious experience corresponds to structured and accessible regions of neural state space rather than to neural activity in general.

## 5 Canonical qualia spaces across modalities

### 5.1 Motivation: sensory systems as natural testbeds

While global measures of neural organization and accessibility provide evidence for structural constraints on conscious states, sensory systems offer particularly clear test cases for examining the relationship between neural representation and experiential structure. In several sensory modalities, qualitative features of experience such as similarity, continuity, and categorical boundaries are well characterized phenomenologically and can be directly compared with neural representations.

These systems therefore serve as canonical examples in which the geometry and topology of experiential space can be empirically investigated, providing concrete instances of how structured neural state spaces correspond to structured conscious experience.

### 5.2 Color perception and opponent-process geometry

Color perception provides one of the most extensively studied examples of structured qualia space. Phenomenologically, colors are experienced as occupying a continuous, multidimensional space characterized by systematic similarity relations and opponent dimensions. Psychophysical studies have long established that perceived color differences can be organized along axes corresponding to opponent channels such as red-green and blue-yellow (Hurvich and Jameson, 1957).

Neurophysiological and neuroimaging studies reveal that these phenomenological structures are mirrored in neural representations within visual cortex. Cortical responses encode color information along opponent dimensions, and multivariate analyses demonstrate that distances between neural activity patterns correspond to perceptual color similarity (Conway and Tsao, 2009; Brouwer and Heeger, 2009). Importantly, color categories emerge as regions within a continuous representational manifold rather than as isolated discrete encodings.

This close correspondence between perceptual geometry and neural state-space organization provides strong evidence that qualitative aspects of visual experience are reflected in the metric structure of neural representations.

### 5.3 Auditory pitch and periodic topology

Auditory pitch perception offers a complementary example of nontrivial experiential topology. Phenomenologically, pitch is experienced as ordered along a continuous dimension, yet it also exhibits periodic structure, as tones separated by octaves are perceived as closely related despite differing in absolute frequency.

Neural recordings in auditory cortex reveal populations selectively tuned to pitch, with response properties consistent with this phenomenology (Bendor and Wang, 2005). Representational analyses suggest that pitch-related neural activity forms continuous trajectories in neural state space, with periodic organization reflecting octave equivalence.

This cyclic topology cannot be captured by simple linear mappings, underscoring the importance of considering topological structure when relating neural representations to conscious experience. Pitch perception thus illustrates how experiential spaces may exhibit non-Euclidean geometry grounded in neural organization.

### 5.4 Somatosensory experience and distorted metric structure

The somatosensory system provides a further example in which experiential geometry is markedly non-uniform. Phenomenologically, different regions of the body are experienced with varying acuity and salience, leading to a distorted representation of bodily space. This distortion is classically illustrated by the somatosensory homunculus, in which cortical representation is disproportionately allocated to regions such as the hands and face (Penfield and Boldrey, 1937).

Neurophysiological and stimulation studies demonstrate that these distortions correspond directly to the organization of somatosensory cortex. Neural state-space representations exhibit expanded regions corresponding to highly sensitive body parts, while other regions are compressed. As a result, distances in neural state space do not map uniformly onto physical distances on the body, but instead reflect experiential relevance.

This mapping highlights that experiential geometry is shaped by neural representational structure rather than by external physical coordinates, reinforcing a structural account of qualia.

### 5.5 Continuity, boundaries, and categorical organization

Across sensory modalities, canonical qualia spaces exhibit a combination of continuity and discreteness. Experiences often vary smoothly along certain dimensions, yet sharp categorical boundaries can emerge, such as between phonemes in speech or basic color categories. Neural state-space analyses reveal corresponding features, with continuous manifolds punctuated by regions of increased separation or reduced transition probability (Kriegeskorte et al., 2008; Haxby et al., 2001).

Such organization suggests that experiential categories arise from the structure of neural representations themselves rather than from arbitrary labeling. From a structural perspective, categorical perception corresponds to regions of relative stability within neural state space, separated by boundaries where small changes in neural activity can produce large changes in experience.

### 5.6 Summary: sensory qualia as structured state spaces

Canonical sensory modalities provide compelling empirical evidence that qualitative aspects of conscious experience correspond to structured neural state spaces. Across vision, audition, and somatosensation, experiential similarity, continuity, categorical boundaries, and distortions are mirrored in the geometry and topology of neural representations.

These cases demonstrate that qualia are not arbitrary or ineffable additions to neural processing, but exhibit systematic structure that can be empirically investigated and compared across modalities. As such, sensory qualia spaces provide a concrete foundation for understanding conscious experience in terms of neural state-space organization and motivate broader synthesis across paradigms, which we pursue in the next section.

## 6 Cross-cutting structural themes in neural states associated with conscious experience

The preceding sections have reviewed diverse experimental approaches bearing on the neural correlates of conscious experience, including contrastive paradigms, representational geometry, perturbational methods, and canonical sensory systems. Although these approaches differ in methodology and scope, they converge on a common conclusion: conscious experience is associated with neural states that exhibit specific, recurring structural properties. This section synthesizes these findings to identify cross-cutting features of neural state space—including restriction, geometry, dimensionality, accessibility, and constrained transitions—that recur across modalities, tasks, and measurement techniques.

### 6.1 From correlates to structure

Across the diverse experimental approaches reviewed in the preceding sections, a consistent pattern emerges: conscious experience is associated not with neural activity in general, but with neural states exhibiting specific structural properties. Neural correlates of consciousness identify restricted subsets of activity patterns; representational geometry reveals organized manifolds and metric relationships; perturbational studies demonstrate selective accessibility and integration; and canonical sensory systems exhibit systematic experiential topologies mirrored in neural representations (Koch et al., 2016).

Taken together, these findings motivate a shift in emphasis from identifying isolated neural correlates to characterizing the structure of neural state space itself. Conscious experience, on this view, is not marked by the presence of particular signals or regions alone, but by participation in structured configurations that satisfy multiple empirical constraints.

### 6.2 State restriction and admissibility

A recurring theme across paradigms is the restriction of conscious experience to a limited subset of possible neural states. Many neural configurations support sensory processing, motor behavior, or cognitive function without being consciously experienced. Only certain configurations reliably correlate with reported experience (Aru et al., 2012).

This restriction is evident in dissociations such as blindsight, anesthesia, sleep, and disorders of consciousness, as well as in contrastive NCC paradigms (Koch et al., 2016; Tamietto and Koch, 2008). From a organization-focused interpretation, these findings suggest that neural state space contains regions that are admissible for experience and others that are not, even though both may be dynamically active.

Importantly, admissibility does not appear to be determined by local activity levels or isolated features, but by global patterns of organization, coordination, and accessibility.

### 6.3 Geometry and experiential similarity

A second cross-cutting theme is the close correspondence between experiential similarity and the geometry of neural representations. Representational similarity analyses consistently show that distances in neural state space track perceptual similarity judgments, while clustering reflects categorical distinctions (Kriegeskorte et al., 2008).

This relationship implies that qualitative features of experience—such as similarity, difference, and category membership—are grounded in the metric structure of neural representations. Experience is thus organized, not merely labeled, by neural state-space geometry, reinforcing the importance of population-level structure over localized signals (Haxby et al., 2001).

### 6.4 Dimensionality reduction and compression

Despite the high dimensionality of neural systems, experiential states consistently exhibit low-dimensional structure. Dimensionality reduction and manifold analyses demonstrate that neural activity associated with perception and cognition occupies constrained subspaces capturing dominant modes of variation relevant to experience (Cunningham and Yu, 2014; Gallego et al., 2017).

This compression helps explain why conscious experience appears ordered and tractable despite underlying biological complexity. It also suggests that experiential structure reflects constraints on neural dynamics that limit the effective degrees of freedom available to conscious states.

### 6.5 Accessibility, integration, and stability

Perturbational approaches highlight an additional structural distinction: conscious states are characterized by dynamic accessibility, integration, and stability under perturbation. Neural states associated with experience support complex, differentiated responses that propagate across distributed networks, whereas unconscious states exhibit reduced reachability and rapid collapse of activity (Casali et al., 2013; Rosanova et al., 2018).

Accessibility thus functions as an empirical criterion distinguishing regions of neural state space capable of sustaining experience. This criterion is orthogonal to representational content and baseline activity, underscoring the importance of dynamical structure in addition to geometric organization. Importantly, low dimensionality alone is not sufficient for conscious experience. Pathological states such as epileptic hypersynchrony may exhibit reduced dimensionality while lacking the differentiated and accessible structure characteristic of conscious conditions.

### 6.6 Continuity, boundaries, and transitions

Across modalities and paradigms, neural state space exhibits a mixture of continuity and discreteness. Experiential dimensions often vary smoothly, yet sharp boundaries can emerge between categories or states. Neural representations reflect this hybrid organization through manifolds punctuated by regions of separation or reduced transition probability (Kriegeskorte et al., 2008; Haxby et al., 2001).

Transitions between experiential states correspond to trajectories through state space that are themselves constrained. Some transitions are gradual and reversible, while others involve abrupt shifts or loss of structure, as observed during anesthesia or sleep onset (Massimini et al., 2012).

### 6.7 Convergence across modalities and methods

A key strength of the structural perspective is its convergence across methods and contexts. Evidence for state restriction, geometric organization, dimensional compression, and accessibility arises from fMRI, EEG, MEG, single-unit recordings, perturbational studies, and clinical observations. Similar structural patterns appear across sensory modalities, cognitive domains, and levels of consciousness (Koch et al., 2016).

This convergence suggests that the identified structural properties are not artifacts of specific paradigms or measurement techniques, but reflect general features of neural organization associated with conscious experience.

### 6.8 Interdependence of structural properties

The five structural properties identified in this review—state restriction, geometric organization, low-dimensional embedding, accessibility, and constrained transitions—are analytically distinguishable but empirically interdependent. State restriction can be understood as defining the bounded region of neural state space associated with conscious conditions. Geometric organization and low dimensionality describe the structural form of that region, while accessibility and constrained transitions characterize its dynamical properties.

In this interpretation, low dimensionality and geometry function primarily as descriptive features of representational organization, whereas accessibility and transition constraints introduce temporal and dynamical criteria. Together, these properties specify not only where neural trajectories tend to reside under conscious conditions, but also how they evolve within a restricted manifold. The ordering presented here is intended as a heuristic clarification rather than a claim of strict logical or metaphysical priority, and serves to organize convergent empirical findings within pluralistic research paradigms.

The structural properties identified here should therefore be interpreted as empirically recurring features associated with conscious states. Whether they are individually necessary, jointly sufficient, or instead reflect partially overlapping correlates remains an open empirical question. The present synthesis does not resolve this issue but delineates the structural terrain on which such determinations must be made.

### 6.9 Distinguishing structural complexity from experiential admissibility

Several of the structural properties discussed in this review—low dimensionality, geometric organization, restricted dynamics, and structured trajectories—also appear in forms of complex cognition that are not necessarily accompanied by conscious experience. Working memory representations, motor planning states, and decision variables can exhibit low-dimensional manifolds and geometry-based organization (Mante et al., 2013; Vyas et al., 2020). Attention may similarly involve constrained transitions within restricted regions of neural state space.

For this reason, no single structural feature identified here should be interpreted as exclusive to consciousness. Rather, the present synthesis concerns the convergent co-occurrence of these properties under experimentally defined conscious conditions—specifically, conditions in which subjective experience is reportable and dynamically accessible under perturbation.

Here, “experiential admissibility” refers operationally to neural states that reliably co-occur with reportable conscious experience in contrastive paradigms or level-of-consciousness manipulations. The distinction between complex cognition and experiential admissibility therefore rests not on dimensionality or geometry alone, but on the joint presence of: (i) restriction to a bounded region of state space, (ii) organized representational geometry tracking experiential similarity, (iii) low-dimensional embedding, (iv) dynamic accessibility under causal perturbation, and (v) constrained but traversable transitions within that region.

In particular, perturbational accessibility provides an important differentiator. Neural states associated with conscious experience tend to exhibit integrated and differentiable responses to external perturbation, maintaining structured propagation across distributed networks rather than collapsing into stereotyped or locally confined activity (Casali et al., 2013; Koch et al., 2016). This reflects not merely structural organization, but the capacity of the system to sustain coordinated, information-rich state transitions following causal intervention.

By contrast, certain forms of complex but non-experiential processing may display geometric structure without demonstrating the same degree of globally integrated, dynamically sustained responsiveness across variations in arousal or consciousness. The claim is therefore not that these properties are individually unique to consciousness, but that their joint presence under perturbationally accessible and reportable conditions forms a recurrent structural profile associated with conscious states.

Whether this co-occurrence is strictly necessary, jointly sufficient, or instead reflects a high-probability empirical clustering remains an open question. The present review delineates the structural pattern suggested by current evidence while leaving its ultimate explanatory status to future empirical investigation.

### 6.10 Summary: toward a structural characterization of conscious states

The findings synthesized here indicate that conscious experience is consistently associated with neural states that are structurally constrained, geometrically organized, low-dimensional, and dynamically accessible. These properties jointly distinguish experiential states from the broader space of neural activity and provide a unifying empirical framework for understanding consciousness without committing to a specific explanatory theory. Table 3 summarizes these structural features.

By focusing on structure rather than mechanism, this perspective clarifies what is currently established about the neural organization of conscious experience and delineates the empirical terrain on which future theoretical and experimental work must operate.

The structural synthesis presented here is intended as a descriptive integration of convergent empirical findings rather than as a proposal of a novel explanatory theory.

**Figure 2:**
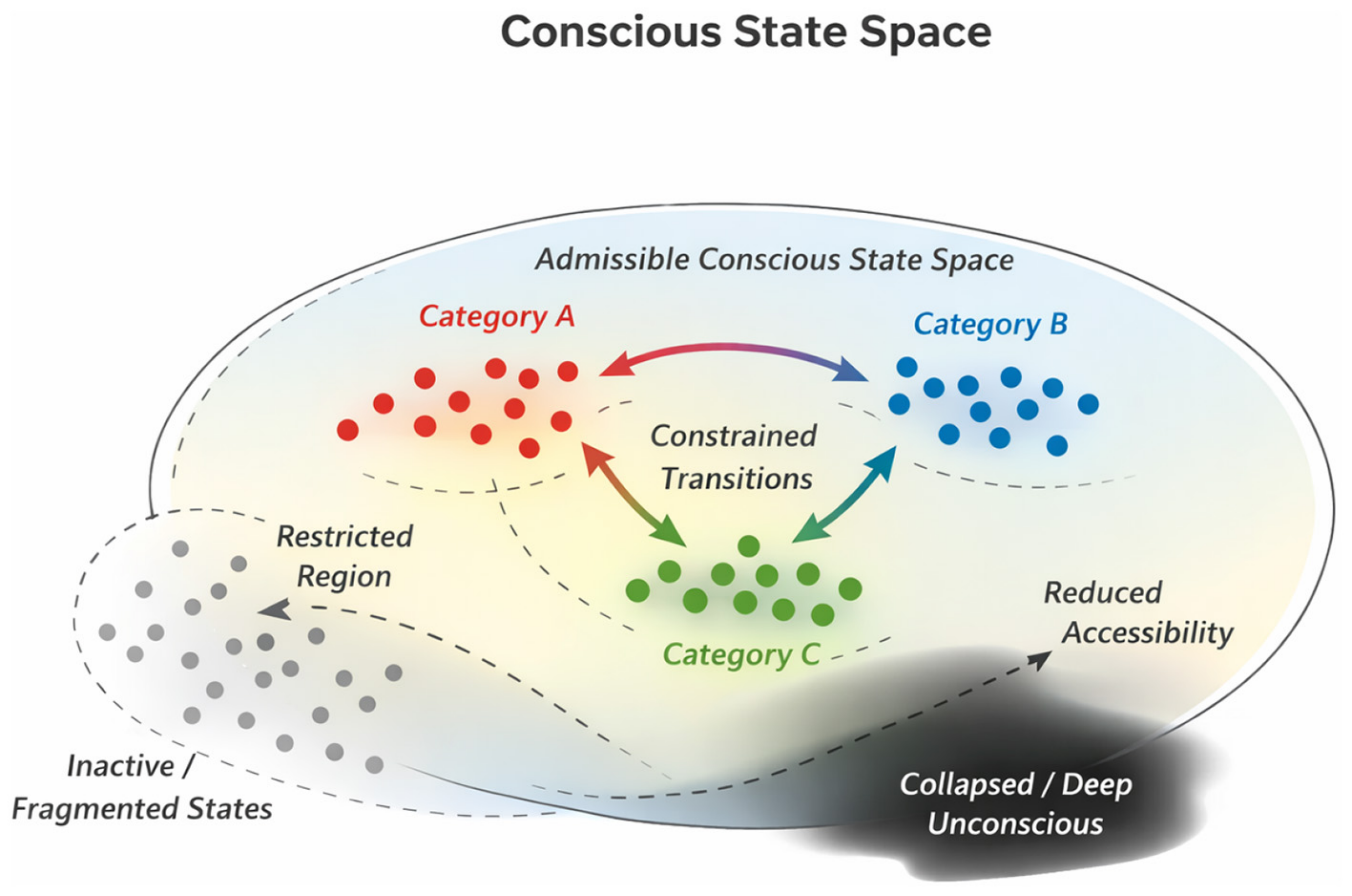
Conceptual schematic of neural state space associated with conscious experience. Neural activity occupies a high-dimensional state space, within which conscious experience is associated with restricted, structured, and dynamically accessible regions. Clusters represent families of neural states corresponding to distinct experiential categories, with distances reflecting representational geometry and similarity relations. Arrows indicate constrained transitions between experiential states, while peripheral or faded regions denote reduced accessibility, fragmentation, or collapse of available state-space structure associated with unconscious conditions such as deep sleep, anesthesia, or disorders of consciousness. The figure is schematic and intended to summarize convergent empirical findings rather than depict a specific mechanistic or theoretical model.

Figure 2 provides a schematic overview of how these properties jointly organize neural state space, illustrating restriction, geometry, accessibility, and constrained transitions. Table 2 summarizes the principal structural properties of neural states associated with conscious experience and the convergent empirical evidence supporting each property. Table 3 provides a complementary summary of key methodological approaches used to characterize neural state-space structure across correlational, geometric, and perturbational paradigms.

**Table 2:** Cross-cutting structural properties of neural states associated with conscious experience and representative empirical evidence supporting each property.

Structural property	Empirical evidence base	Key implication for experience
State restriction admissibility	Contrastive NCC paradigms; blindsight; anesthesia; disorders of consciousness	Only a subset of neural configurations supports conscious experience
Geometric organization	Representational similarity analysis; multivariate decoding; sensory feature spaces	Experiential similarity and difference reflect distances in neural state space
Low-dimensional structure	Neural manifold studies; dimensionality reduction across tasks and modalities	Conscious experience occupies constrained subspaces of neural activity
Accessibility and integration	TMS–EEG perturbation; perturbational complexity index; recovery of consciousness	Experiential states must be dynamically reachable and internally integrated
Constrained transitions	Perceptual switching; sleep and anesthesia transitions; state-space trajectories	Changes in experience follow structured paths rather than arbitrary jumps

**Table 3:** Mapping empirically motivated structural properties of conscious neural states onto major theoretical frameworks.

Structural property	Global workspace	Integrated information theory (IIT)	Predictive processing
State restriction/admissibility	Selective broadcasting and gatekeeping determine which representations enter the workspace	Exclusion of states with insufficient integration from the maximally irreducible conceptual structure	Precision weighting suppresses low-confidence or prediction-inconsistent states
Low dimensionality	Not a central theoretical commitment, but compatible with compression of broadcast content	Central role of low-dimensional conceptual space defined by integrated information	Latent state compression in hierarchical generative models
Geometric organization	Implicit organization via representational similarity and workspace competition	Explicit geometric structure of conceptual space	Representational geometry shaped by priors and prediction-error minimization
Accessibility and reachability	States must be broadcast-ready to influence global cognition	States must belong to an integrated repertoire accessible to the system	States must be dynamically reachable under precision-weighted inference
Constrained transitions	Competition and updating within the workspace limit abrupt state changes	Transitions constrained by changes in integrated conceptual structure	Belief updating follows structured trajectories in state space

The table is descriptive and intended to illustrate points of contact rather than adjudicate between theories.

Importantly, convergence at the level of structural constraints does not imply theoretical equivalence, but suggests that diverse accounts may be responding to common empirical regularities in neural organization.

## 7 Methodology and conceptual challenges

### 7.1 Structural interpretation of electrophysiological state-space organization

Electrophysiological recordings obtained via EEG and MEG reveal that neural dynamics occupy a richly structured yet highly non-uniform region of state space. Although neural activity evolves continuously in time, empirical reconstructions consistently demonstrate clustering, sparsity, and trajectory-dependent organization rather than uniform exploration of available configurations. The state-space structure identified in the present analysis can be interpreted in terms of electrophysiological constraints that shape which regions of configuration space are dynamically accessible and structurally stable.

A first distinction concerns *transiently coherent regions* of state space. Neural activity frequently exhibits brief episodes of local or regional phase alignment, reflected in short-lived increases in phase-locking value or narrow-band oscillatory bursts. In reduced state-space representations, such dynamics correspond to shallow or weakly populated regions that are revisited frequently but not persistently occupied. These regions contribute to the fine-scale texture of the state space without forming robust clusters, consistent with their limited temporal stability and lack of long-range coordination (Melloni et al., 2007; Siegel et al., 2012).

A second class corresponds to *diffuse or weakly structured regions* associated with incoherent background dynamics. These trajectories exhibit low global synchrony, broad spectral features, and high temporal variability. In embedded representations, such activity appears as dispersed, low-density regions that form the background against which more structured trajectories are organized. Importantly, these regions are not devoid of activity; rather, they reflect configurations that fail to stabilize into coherent geometrical features despite ongoing neural dynamics (Deco and Jirsa, 2011; Breakspear, 2017).

More structured regions emerge through *trajectory-history–dependent organization*. Following transitions between metastable configurations, residual patterns of phase organization may persist and bias subsequent trajectories through state space. Empirically, such effects manifest as hysteresis, asymmetries in transition probabilities, and non-Markovian structure in time-embedded neural dynamics. In mapped state spaces, this appears as curved manifolds or preferential pathways linking otherwise distinct clusters, reflecting the influence of prior dynamic.

### 7.2 Measurement limitations and indirect access to experience

A fundamental challenge in the scientific study of conscious experience is that experience itself is not directly observable. Empirical investigations must therefore rely on indirect measures, including behavioral reports, physiological proxies, and neural signatures inferred from experimental paradigms (Overgaard, 2018). Although advances such as no-report paradigms and clinical assessments reduce reliance on explicit reporting, all current approaches involve some degree of inference.

This limitation constrains the interpretation of structural findings. Neural state-space organization can be characterized with increasing precision, but the mapping between neural structure and subjective experience remains mediated by experimental assumptions. Consequently, robust conclusions regarding experiential structure require convergence across multiple paradigms and measurement modalities rather than reliance on any single approach (Koch et al., 2016).

### 7.3 Report dependence and task confounds

Many studies of conscious experience require subjects to report perceptual content, confidence, or awareness, introducing potential confounds related to attention, decision-making, and motor preparation. Neural activity associated with these processes may overlap with or obscure activity related to experience itself (Aru et al., 2012).

No-report paradigms address some of these concerns by inferring perceptual content from physiological or stimulus-driven markers rather than explicit responses. However, these paradigms introduce their own assumptions and may not fully eliminate task-related influences (Boly et al., 2017). From a structural perspective, disentangling experiential states from report-related states remains a central methodological challenge.

### 7.4 Individual variability and cross-subject alignment

Neural representations exhibit substantial variability across individuals, reflecting differences in anatomy, development, and experience. This variability complicates efforts to define common neural state spaces or to align representational geometries across subjects. Techniques such as hyperalignment and shared response modeling improve cross-subject correspondence, but they introduce additional modeling assumptions and preprocessing steps (Haxby et al., 2001).

Individual differences may also extend to experiential structure itself, raising questions about the universality of inferred qualia spaces. Structural regularities identified at the group level may obscure meaningful subject-specific organization. Addressing this tension requires careful balance between aggregation for statistical power and sensitivity to individual variation.

### 7.5 Temporal resolution and state definition

Conscious experience unfolds dynamically, yet many experimental paradigms rely on discretized time windows or averaged responses. Defining what constitutes a neural “state” therefore depends on choices regarding temporal granularity, segmentation, and analysis window (Cunningham and Yu, 2014).

Different measurement modalities offer complementary temporal resolutions, but integrating these data into a unified state-space framework remains challenging. Transient or rapidly evolving experiential states may be difficult to capture with standard experimental designs, limiting insight into fine-grained transitions and short-lived structures.

### 7.6 Interpretation of dimensionality and manifold structure

Dimensionality reduction and manifold learning techniques are central to identifying structure in neural data, but their outputs depend on algorithmic choices, preprocessing, and noise characteristics. Different methods may yield different embeddings, raising questions about robustness and interpretability (Cunningham and Yu, 2014; Gallego et al., 2017).

While low-dimensional structure is a recurring empirical finding, its relationship to underlying neural mechanisms and to experience is not always straightforward. Reduced representations should therefore be interpreted as descriptive tools rather than as literal mappings of biological organization.

### 7.7 Limits of perturbational inference

Perturbational approaches provide valuable insights into accessibility and integration, but they also have limitations. Noninvasive perturbations such as TMS are spatially coarse and may engage network-level effects beyond the targeted region. Clinical stimulation studies offer higher spatial precision but are constrained by ethical and practical considerations (Casali et al., 2013; Massimini et al., 2012).

Moreover, while perturbation reveals properties of neural organization, it does not by itself establish causal explanation in a strong sense. Perturbational findings must therefore be interpreted in conjunction with correlational and geometric analyses.

### 7.8 Scope and generalization

Finally, questions remain regarding the generalizability of structural findings across species, developmental stages, and altered states of consciousness. Animal models provide valuable insights into neural organization, but the absence of direct report complicates interpretation. Pharmacologically induced altered states may reveal novel structures but also introduce confounding factors (Koch et al., 2016).

A comprehensive structural account of conscious experience will need to integrate findings across these domains while remaining sensitive to context-specific constraints.

### 7.9 Summary: constraints as opportunities

The challenges outlined above underscore the complexity of studying conscious experience, but they also highlight opportunities for methodological innovation. By explicitly acknowledging measurement limits, individual variability, and analytic assumptions, future research can refine structural descriptions of neural states and strengthen the empirical foundations of consciousness science.

Addressing these challenges will be essential for advancing from descriptive synthesis toward more precise characterization of the neural organization associated with conscious experience.

## 8 Implications and future directions

The structural synthesis developed in this review indicates that conscious experience is associated not merely with neural activation, but with neural states exhibiting specific organizational properties, including restriction to admissible configurations, low-dimensional structure, meaningful geometry, and dynamic accessibility. While the preceding sections have emphasized convergent empirical evidence for these properties, an important next step is to examine how this structural perspective can be operationalized in experimentally testable ways.

One implication of the state-space framework is that conscious and unconscious conditions should differ not only in overall activity patterns, but in the geometry, dimensionality, and accessibility of neural state space. If conscious states occupy low-dimensional, dynamically reachable manifolds, then perturbations may induce structured state transitions that remain constrained to manifold boundaries during conscious states, while producing fragmented, unstable, or rapidly collapsing trajectories during unconscious conditions such as deep anesthesia or non-REM sleep. This possibility can be investigated using perturbation-based paradigms combined with state-space reconstruction (Massimini et al., 2012; Tononi et al., 2016).

Advances in recording and stimulation technologies—including higher-density electrophysiology, improved temporal resolution, and multimodal integration across fMRI, EEG, MEG, and invasive recordings—provide increasing capacity to reconstruct neural state spaces with sufficient fidelity to examine such structural differences across modalities and individuals (Cunningham and Yu, 2014; Siegel et al., 2015).

A related implication concerns the distinction between activation and accessibility. Neural states with comparable levels of activation may differ in their experiential correlates depending on whether they are reachable from neighboring states under perturbation or ongoing dynamics. This suggests that measures of reachability, transition probability, or integration could, in some contexts, provide more discriminating indicators of conscious state organization than static activation metrics alone (Koch et al., 2016; Boly et al., 2017). Perturbational and dynamical analyses may therefore complement correlational approaches in refining empirical criteria for conscious states.

Temporal analyses offer an additional avenue for investigation. If loss of consciousness involves a collapse, fragmentation, or reduction in accessible regions of neural state space, then measurable changes in effective dimensionality or transition structure may precede behavioral unresponsiveness during anesthesia induction or sleep onset. Longitudinal and sliding-window analyses provide methodological tools for assessing whether structural reorganization anticipates phenomenological change rather than merely accompanying it (Sanders et al., 2012; Pigorini et al., 2015).

Extending structural analyses beyond canonical sensory domains to emotion, memory, imagery, and abstract cognition will be important for evaluating the generality of these empirical patterns across diverse forms of conscious experience.

Beyond global state changes, convergent findings raise the possibility of cross-modal invariance in structural constraints. Despite differences in representational content across sensory modalities, conscious states in vision, audition, and somatosensation may share common geometric and accessibility properties. Individual differences, in turn, might reflect systematic variations or deformations within a broadly shared state-space architecture rather than fundamentally distinct organizational principles.

Together, these considerations outline a pathway from descriptive synthesis toward increasingly precise empirical tests. By emphasizing falsifiable structural consequences rather than specific mechanistic commitments, future research can assess whether the organizational properties identified here are necessary, jointly sufficient, or merely correlated with conscious experience, thereby sharpening the empirical foundations of consciousness science.

## 9 Conclusion

This review has synthesized empirical findings from neural correlates of consciousness research, representational geometry, perturbational approaches, and canonical sensory systems to examine the structural properties of neural states associated with conscious experience. Across paradigms, modalities, and measurement techniques, a consistent pattern emerges: conscious experience is reliably associated with neural states that are restricted, organized, low-dimensional, and dynamically accessible.

Rather than identifying isolated neural markers or mechanisms, the reviewed evidence supports a structural characterization of conscious states. Neural activity supporting experience occupies constrained regions of neural state space, exhibits metric relationships that track experiential similarity, and remains accessible and stable under perturbation. These properties jointly distinguish experiential states from the broader landscape of neural dynamics.

By focusing on structure rather than explanation, this review clarifies what is currently established about the neural organization of conscious experience while remaining agnostic about underlying mechanisms or ontological commitments. The resulting synthesis delineates a set of empirical constraints that any comprehensive account of consciousness must accommodate, and it highlights promising directions for future research aimed at refining structural descriptions, improving measurement, and integrating across paradigms.

As the neuroscience of consciousness continues to mature, structural approaches offer a way to consolidate diverse findings into a coherent empirical program. Characterizing the geometry, topology, and accessibility of neural state space may not by itself resolve longstanding conceptual questions, but it provides a stable and testable foundation on which more explanatory and interventional advances can be built.
